# A Newly Developed Exergame-Based Telerehabilitation System for Older Adults: Usability and Technology Acceptance Study

**DOI:** 10.2196/48845

**Published:** 2023-12-07

**Authors:** Julia Seinsche, Eling D de Bruin, Enrico Saibene, Francesco Rizzo, Ilaria Carpinella, Maurizio Ferrarin, Sotiria Moza, Tanja Ritter, Eleftheria Giannouli

**Affiliations:** 1 Movement Control and Learning Group, Institute of Human Movement Sciences and Sport Department of Health Sciences and Technology ETH Zurich Zurich Switzerland; 2 Department of Health OST - Eastern Swiss University of Applied Sciences St. Gallen Switzerland; 3 Department of Neurobiology, Care Sciences and Society Karolinska Institutet Huddinge Sweden; 4 Istituto di Ricovero e Cura a Carattere Scientifico Fondazione Don Carlo Gnocchi Onlus Milan Italy; 5 Materia Group Nicosia Cyprus; 6 Division of Sports and Exercise Medicine Department of Sport, Exercise and Health University of Basel Basel Switzerland

**Keywords:** older adults, motor-cognitive intervention, exergame, telerehabilitation, information and communications technologies, user-centered design, usability, technology acceptance

## Abstract

**Background:**

Telerehabilitation has gained significance as a tool to deliver and supervise therapy and training as effective as traditional rehabilitation methods yet more accessible and affordable. An exergame-based telerehabilitation system has recently been developed within the scope of the international Continuum-of-Care (COCARE) project. The system comprises training devices for use in clinics (Dividat Senso) and at home (Dividat Senso Flex), an assessment system, and a rehabilitation cockpit, and its focus lies on home-based motor-cognitive training, which is remotely managed by health care professionals (HPs).

**Objective:**

This study aims to analyze the usability, acceptance, and enjoyment of the COCARE system from the perspective of primary (older adults [OAs]) and secondary (HPs) end users.

**Methods:**

At 3 trial sites (located in Switzerland, Italy, and Cyprus), participants engaged in a single-session trial of the COCARE system, including testing of exergames and assessments. Mixed methods encompassing qualitative approaches (eg, think aloud) and quantitative measures (eg, Exergame Enjoyment Questionnaire [EEQ], System Usability Scale [SUS], and Unified Theory of Acceptance and Use of Technology [UTAUT] questionnaire) were used to analyze participants’ perceptions of the system and identify potential barriers to its implementation in a home setting. In addition, the associations of performance during gameplay and assessments, demographics, and training motivation (Behavioral Regulation in Exercise Questionnaire–3 [BREQ-3]) with usability, acceptance, and enjoyment were explored.

**Results:**

A total of 45 OAs and 15 HPs participated in this study. The COCARE system achieved good acceptance ratings (OAs: 83%, range 36%-100% and HPs: 81%, range 63.8%-93.3% of the maximum score), and OAs indicated high enjoyment (mean 73.3, SD 12.7 out of 100 points in the EEQ) during the exergame session. The system’s usability, assessed with the SUS, received scores of 68.1 (SD 18.8; OAs) and 70.7 (SD 12.3; HPs) out of 100 points, with substantial differences observed between the trial sites. Several requirements for improvement were identified. Commonly mentioned barriers to adoption included the movement-recognition sensitivity of the Senso Flex, its limited markings, and difficulties in understanding certain instructions for assessments and games. Performance in games and assessments showed the highest significant correlations with the SUS (Spearman ρ=0.35, *P*=.02 to ρ=0.52, *P*<.001). The BREQ-3 had significant correlations with all usability measures, thereby even large significant correlations with enjoyment (Spearman ρ=0.58; *P*<.001). Age had moderately significant correlations with the SUS (Spearman ρ=−0.35; *P*=.02) and the UTAUT total score (ρ=−0.35; *P*=.02) but no significant correlation with the EEQ. Concerning sex and years of education, no significant correlations were found.

**Conclusions:**

The study’s findings will inform the further development of the COCARE system toward a user-friendly and widely accepted version, enhancing cognitive and physical functions in OAs. Future randomized controlled trials should evaluate the system’s feasibility and effectiveness.

## Introduction

### Background

In recent decades, the development of health technology systems to support patients and health care professionals (HPs) has increased dramatically. For instance, information and communications technologies (ICTs) have recently emerged as valuable tools for telerehabilitation in older adults (OAs) and various patient groups. Telerehabilitation can be defined as the delivery of rehabilitation services from a distance using ICTs [1] and includes home-based technology-assisted training as well as a digital centralized remote management of this training [2]. In this way, OAs are able to independently perform cognitive, physical, or other forms of training in their home environment while being guided remotely by HPs [3]. Consequently, telerehabilitation holds promise as a cost-effective solution to meet the growing demand for health services because of population aging and the increasing service costs for usual care [4].

An emerging training approach that lends itself to telerehabilitation is the use of exergames (ie, interactive video games that combine motor and cognitive tasks [5]). Previous research and evidence from systematic reviews suggest that simultaneous motor and cognitive training may be superior to separate and possibly even to sequential training of both functions [6-12]. Indeed, exergames have been shown to yield improvements in several physical functions, including lower-extremity muscle strength [13], dual-task walking speed [13,14], step reaction time [14], balance [13,15-17], and aspects of gait [18]. In addition, exergames have demonstrated positive effects on cognitive functions such as reaction time in cognitive tasks [13], executive functioning [13,19,20], short-term attentional span, processing speed [18], exercise enjoyment [21], and health-related quality of life [22,23].

Although popular exergame systems such as Nintendo Wii or Xbox Kinect exist, they were not purpose developed for training OAs, potentially overlooking their unique needs. An alternative solution is the Continuum-of-Care (COCARE) system (Dividat), an exergame-based telerehabilitation system designed to meet the specific needs and requirements of OAs. Overall, the system comprises an exergame-based training tool, an assessment system, and a centralized digital case manager (rehabilitation cockpit).

To ensure the usability, feasibility, and effectiveness of new technologies for rehabilitation, a user-centered design (UCD) approach is essential. UCD is defined as an iterative design process involving end users at every stage of a research and development project. This approach facilitates a comprehensive understanding of the factors influencing the use of the corresponding technology and ensures that this technology is acceptable, purposeful, usable, safe, and effective [24,25]. A UCD is particularly important in technologies developed for OAs considering their unique needs, barriers, and preferences regarding the adoption of ICTs and gaming, which differ from those of younger people [26]. Recently, focus groups were conducted with potential primary (OAs) and secondary (HPs) end users of the COCARE system as a first step toward developing a highly user-friendly design. Participants showed a general interest in ICT-based telerehabilitation but also expressed concerns, particularly regarding ICT literacy, the system’s ease of use, and loss of face-to-face contact with HPs [27]. Therefore, subsequent development efforts focused on simplifying the user interface (UI) and updating the software and hardware of the device for home-based exergame training.

As the next and central step in the UCD process, a usability study was conducted. Usability is defined as “the extent to which a product can be used by specific users to achieve specified goals with effectiveness, efficiency, and satisfaction in a specified context of use” (ISO 924-11) [28]. This definition indicates that acceptance and enjoyment (and safety) are essential components of usability and, therefore, should also be investigated [29].

### Objectives

Thus, the primary aim of this study was to assess the usability, acceptance, enjoyment, and safety of the modified COCARE system for OAs (primary end users) and HPs (secondary end users) and identify facilitators of and barriers to its implementation at home. In addition, the study aimed to analyze potential associations between usability measures and OAs’ performance during gameplay and assessments (eg, total exergame scores and reaction time), demographics, and training-related motivational factors.

## Methods

### Materials

**Figure 1:**
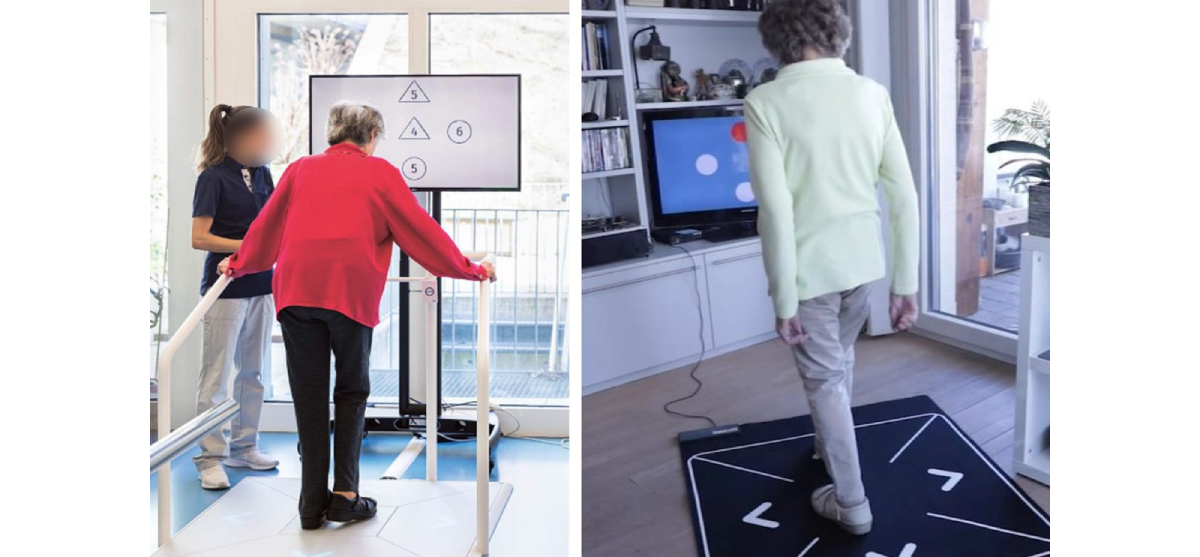
Dividat Senso (left) and Senso Flex (right). Informed consent was obtained from the individuals in the picture allowing for the use of the picture for publication.

**Figure 2:**
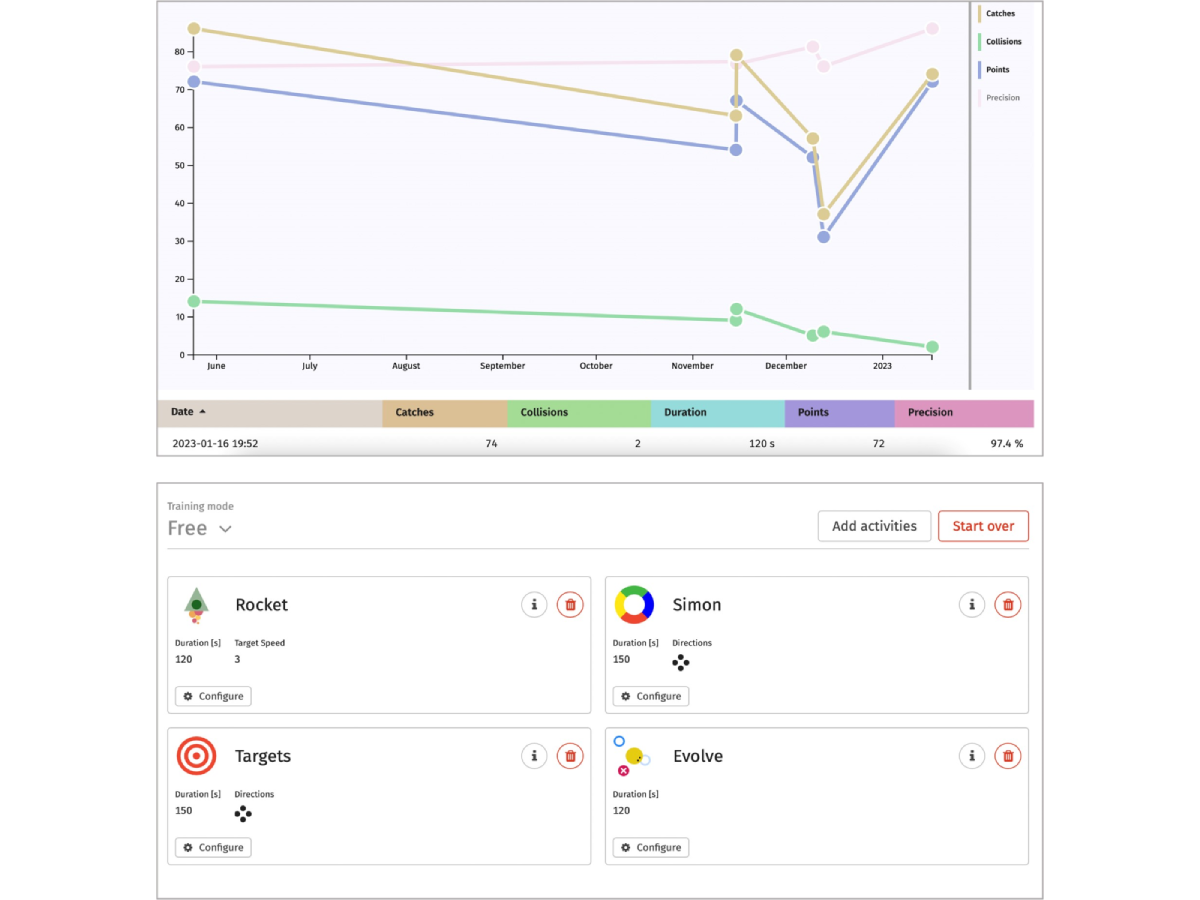
Training overview and management in the rehabilitation cockpit.

The COCARE system as an exergame-based telerehabilitation tool consists of four subsystems: (1) Dividat Senso (Figure 1, left panel), (2) Dividat Senso Flex (Figure 1, right panel), (3) an assessment system, and (4) a rehabilitation cockpit (Figure 2). Dividat Senso is a stepping platform consisting of 5 plates with 4 force measurement sensors per plate and is connected to a 2D screen. Recently, a lighter version (the Dividat Senso Flex) was developed for independent training at home. In both devices, the stimuli of the exergames appear on the screen, and the games are played by stepping in 1 of 4 directions (front, right, left, and back), shifting the body weight, and marching on the middle plate. Thus, the exergames enable the simultaneous training of motor and cognitive functions.

**Figure 3:**
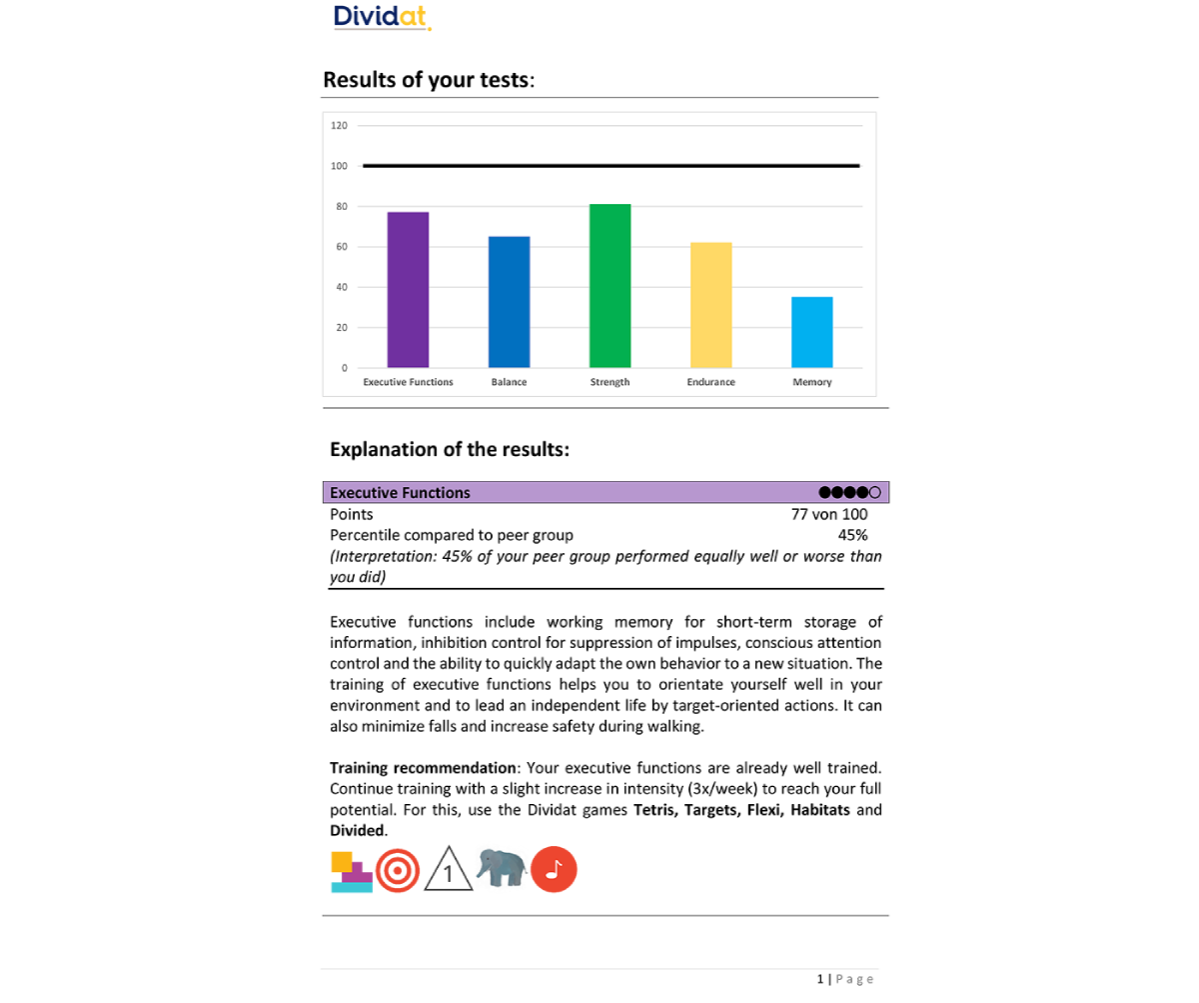
Example assessment report.

The assessment system allows for a comprehensive analysis of a user’s functional status to generate training recommendations. A report on the assessment results is delivered directly to the HPs and to OAs (for the latter, see Figure 3). Subsequently, the rehabilitation cockpit—a digital web-based system—can be used for comprehensive case management, including registration of new patients, scheduling of training sessions, training control, and data monitoring. Further details about the Senso [14,16] and the COCARE system [27] have been described elsewhere.

### Study Design

This usability study was conducted as a cross-sectional study at 3 study sites (ETH Zürich, Switzerland; Materia Group, Cyprus; and Istituto di Ricovero e Cura a Carattere Scientifico, Fondazione Don Carlo Gnocchi, Italy) using a mixed methods design (ie, qualitative [think-aloud method and open questions] and quantitative [questionnaires, game performance, and assessment results] data were collected). We followed the STROBE (Strengthening the Reporting of Observational Studies in Epidemiology) checklist [30] to report this cross-sectional study (Multimedia Appendix 1).

### Ethics Approval

The Ethics Commission of ETH Zürich (EK 2021-N-183); the Ethical Committee of Istituto di Ricovero e Cura a Carattere Scientifico, Fondazione Don Carlo Gnocchi, Milan (ID 05_09/12/2021); and Cyprus National Bioethics Committee (ΕΕΒΚ/ΕΠ/2021/51) examined and approved the study confirming that it complies with the principles of the Helsinki Declaration.

### Participants

We aimed to recruit 20 participants (15 OAs and 5 HPs) at each site, with a total sample size of 60 participants. To determine sample sizes, we considered studies recommending 3 to 5 [31], 10 (–2 to +2) [32], or even 20 participants [33] for usability studies. In the absence of comparable studies, sample size considerations for OAs were based on the 10 (–2 to +2) rule of thumb also taking possible dropouts into account, whereas sample size considerations for HPs, who were less the focus of this study, were in accordance with articles by Virzi [34] and Lewis [31], who proposed the 3 to 5 participants rule.

The inclusion criteria for OAs were (1) age of ≥60 years, (2) being community dwelling, and (3) being physically able to independently stand for at least 2 minutes. The exclusion criteria for OAs were (1) sensory impairments interfering with the use of the system, (2) a Mini-Mental State Examination (MMSE) score of <20, (3) terminal illnesses, and (4) previous or current major psychiatric illnesses. HPs were required to be actively involved in conducting physical or cognitive therapy sessions with older people as part of their workplace role and be registered and accredited members of the health care community.

The recruitment methods in Switzerland included contacting older participants from previous studies of the Motor Control and Learning Group at ETH who had consented to be listed as potential future participants and using ongoing research collaborations with the VAMED rehabilitation center in Dussnang (Switzerland) to recruit HPs. In Cyprus and Italy, participants were recruited via convenience sampling—in Italy of patients usually attending the Fondazione Don Carlo Gnocchi clinics. Potentially eligible OAs and HPs were contacted and informed comprehensively about the study by phone or by handing out or sending flyers, as well as through detailed information sheets.

Recruitment began in January 2022 and continued throughout the trial period, lasting from early February 2022 to late March 2022.

### Study Procedure

Each participant underwent a single assessment session lasting approximately 90 minutes. During this session, the COCARE system components were presented to participants, who subsequently tried them out. Each session at each site followed a standardized protocol corresponding to the natural flow of the COCARE system.

First, the participant’s functional status was assessed using 2 tests on the Senso, beginning each test with a brief warm-up for familiarization before proceeding with the main assessment.

**Figure 4:**
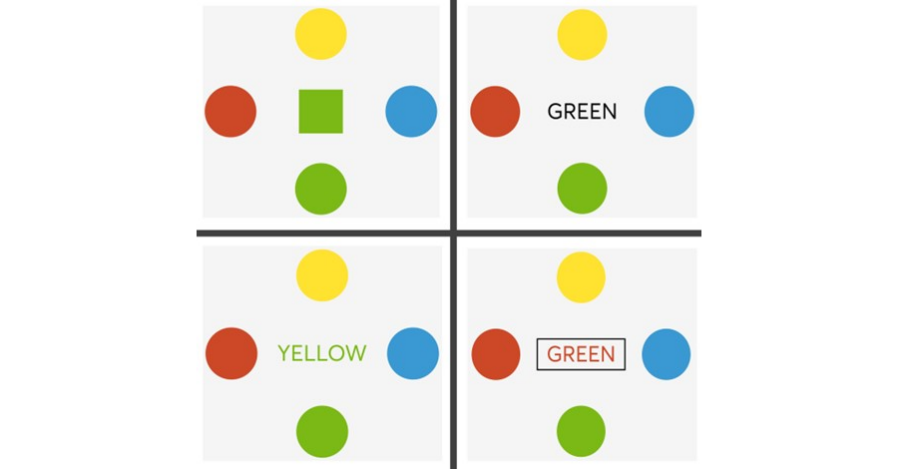
The 4 levels of the Stroop Test (from top left to bottom right).

The first assessment, the Stroop Test, is based on the Color-Word Interference Test by Stroop [35] and consists of 4 trial levels (Figure 4). Throughout all levels, 4 circles with different colors (red, green, blue, and yellow) are shown around the center of the screen. During the individual levels, different stimuli are presented in the center, which the participant then has to match to the appropriate circle with a step. The stimuli in the four levels are as follows:

1. Color part: squares in 4 different colors are displayed in the middle, and the color of the square has to be matched with the color of the respective circle.
2. Word part: the given stimuli are the 4 different colors written in black. The written color has to be matched with the respective circle.
3. Inhibition part: words are written in colors (red, green, blue, and yellow). The color of the writing has to be matched with the circles.
4. Flexibility part: colored words appear in the center. Participants have to switch between selecting the color of the writing and the color they read in case the word is enclosed within a box.

**Figure 5:**
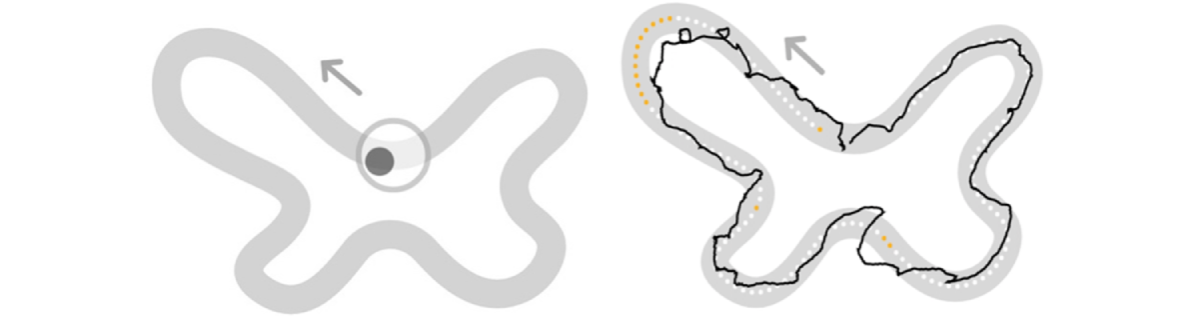
Output of the Coordinated Stability Test.

The second assessment, the Coordinated Stability Test originally developed by Lord et al [36], is designed to measure dynamic balance. Participants were instructed to stand on the middle plate of the Senso with their arms crossed in front of their chest. They then had to shift their center of pressure following a figure displayed on the screen (Figure 5).

HPs performed both assessments twice—first assuming the role of a patient and then acting as a therapist guiding the investigator, who simulated the role of a patient.

Subsequently, the investigators demonstrated the newly adapted UI of the assessment system, which participants had the opportunity to try out. Following this, an example assessment report describing and explaining the participants’ functional status and providing derived training recommendations was presented to all participants.

Afterward, participants were instructed by the investigator to set up the Senso Flex before they engaged in a selection of predetermined exergames on the Senso Flex for 80 to 150 seconds each. The games included the following:

1. Targets (divided attention and action planning; 80 s): balls come flying simultaneously from different directions and need to be hit when reaching the middle of 1 of 4 targets displayed on the screen by stepping in the corresponding direction.
2. Tetris (action planning, visuospatial orientation, and mental rotation; 150 s): differently shaped pieces descending from the top to the bottom have to be rotated and moved to create complete horizontal lines.
3. Rocket (endurance; 80 s): participants control a rocket flying through space by marching on the middle plate. A green arrow and a red bar indicate the need to increase or decrease stepping frequency, respectively.
4. Evolve (balance control, weight shifting, and action planning; 80 s): blue rings, red dots, and a yellow figure are displayed on the screen. Participants control the yellow figure by shifting their center of pressure to catch the blue rings while avoiding the red dots.
5. Simon (short-term memory and memory span; 80 s): a given stepping sequence must be memorized and repeated.

Finally, participants were introduced to the COCARE rehabilitation cockpit, which involved the following two components:

1. A training overview for HPs to monitor adherence displaying the user’s training frequency, components, and performance (Figure 2)
2. The management system, which enables HPs to create a training plan by selecting appropriate games and setting training parameters for each game, such as duration, speed, and other game-specific setting options (Figure 2)

Participants were also informed about the concept of a communication tool integrated into the COCARE system, and their wishes and expectations regarding such a tool were elicited.

Before concluding the session, participants completed questionnaires addressing various aspects of usability, acceptance, and enjoyment.

### Outcome and Outcome Measures in OAs

#### Primary Outcomes

##### Usability

Usability was assessed quantitatively using the System Usability Scale (SUS) [37,38], a validated and reliable instrument for the analysis of the usability of newly developed devices and systems. It is based on 10 items rated on a 5-point Likert scale (from 0=strongly disagree to 4=strongly agree). The total score is calculated by summing all item scores and then multiplying the result by 2.5. Higher scores indicate better usability, and a SUS score of ≥70 is considered “acceptable” [39].

For the qualitative analysis of usability, a usability protocol was created consisting of 5 categories (Dividat Senso, assessment system, Dividat Senso Flex, exergames, and rehabilitation cockpit) that incorporated observations by the investigators and feedback from the participants. Participants were prompted to “think aloud” [40] while testing all components of the COCARE system, and their verbalized thoughts were noted by the investigator. In addition, a self-constructed questionnaire was used to assess the perceived usability of the single components of the COCARE system, addressing, for instance, aspects related to the assessment system’s feasibility, the understanding of each assessment, and the comprehensibility of the assessment report. Furthermore, questions pertaining to the Senso Flex and the rehabilitation cockpit sought participants’ opinions on the games, instructions, hardware, UI, communication ideas, adherence monitoring, and management possibilities. Participants responded on a 5-point Likert scale (1=strongly disagree; 5=strongly agree).

##### Acceptance

Adopting the definition of Peek et al [41], technology acceptance refers to the intention to use a technology or the actual technology use. In this study, acceptance analysis used a questionnaire based on the Unified Theory of Acceptance and Use of Technology (UTAUT) [42], which is an extension of the commonly used Technology Acceptance Model (TAM) [43]. Both the UTAUT and TAM are common approaches in the field of technology acceptance [44]. In contrast to the TAM, the UTAUT not only encompasses factors such as *perceived ease of use* and *perceived usefulness* but also acknowledges that contextual elements (social influences and facilitating conditions) may influence the *behavioral intention to use* (ie, acceptance) or technology adoption [26,45]. Therefore, according to Venkatesh et al [42], the UTAUT can explain up to 70% of the intention to use [42,46]. In this study, the UTAUT questionnaire was created based on previous studies’ measures of its key constructs (*perceived ease of use*, *perceived usefulness*, *social influences*, and *facilitating conditions*) [45]. In addition, the category *attitude*, which has been recognized as another important factor, for instance, in the TAM, was included [26,45]. The evaluation is based on a 5-point Likert scale ranging from 1 (strongly disagree) to 5 (strongly agree). Negatively formulated questions were reverse coded for analysis. The total UTAUT score was obtained by summing all item scores, and subscale scores were calculated using the mean value of each item.

##### Enjoyment

Enjoyment was measured using the Exergame Enjoyment Questionnaire (EEQ) [47], which comprises 20 questions answered on a 5-point Likert scale ranging from 1 (strongly disagree) to 5 (strongly agree). Negatively phrased questions are scored in reverse. This results in a minimum score of 20 points and a maximum score of 100 points.

##### Perceived Safety

The analysis of safety involved questions about dizziness or pain experienced during training. Moreover, critical moments such as tripping, slipping, swaying, or fear of falling were noted in the observation protocol.

#### Secondary Outcomes: Performance Parameters

One performance parameter for each exergame and assessment stored in the rehabilitation cockpit was collected for further analysis.

#### Contextual Factors

The following factors, previously suggested to be determinants of OAs’ perceived usability and acceptance of technological (training) devices [45,48,49], were also included in the analysis:

1. Demographics (age, sex, and years of education)
2. Training motivation, assessed using the Behavioral Regulation in Exercise Questionnaire–3 (BREQ-3) [50-52]. The BREQ-3 is based on the self-determination theory and measures different types of exercise motivation as a multidimensional construct. It comprises 6 subscales (amotivation, as well as external, introjected, identified, integrated, and intrinsic regulation), each consisting of 4 items rated on a 5-point Likert scale ranging from 0 (not true for me) to 4 (very true for me). Mean scores for each subscale and a unidimensional index called the relative autonomy index weighting these mean values were calculated [52,53]. Higher positive scores indicate a stronger overall motivational orientation.

### Outcomes and Outcome Measures in HPs

The outcomes and outcome measures for HPs were similar to those for OAs, with only slightly differing questions. While questions for OAs focused particularly on the comprehensibility of all components, HPs were also asked about their acceptance of the system as part of their therapies. Furthermore, exergame enjoyment, performance measures, and training motivation were omitted as they do not significantly contribute to the system’s usability from a therapist’s perspective.

### Statistical Analysis

Potential differences in demographics between the different trial sites were tested using a 1-way ANOVA for continuous variables and a chi-square test for dichotomous variables.

To quantitatively assess usability, descriptive statistics were generated for all quantitative data resulting from the primary outcomes (SUS, self-made usability questionnaire, UTAUT questionnaire, and EEQ), secondary outcome measures (assessment and performance measures), and contextual factors (demographic factors and BREQ-3).

A bivariate correlation analysis among quantitative usability outcome measures (SUS, UTAUT questionnaire, and EEQ) and secondary as well as contextual factors was conducted using the Spearman correlation coefficient. The level of significance was set at α≤.05 (2-sided). Effect sizes were interpreted as small (*r*<0.30), medium (0.30≤*r*<0.5), and large (*r*≥0.50) [54].

All quantitative statistical analyses were performed using SPSS (version 26; IBM Corp).

## Results

### Primary End Users (OAs)

#### Demographics (OAs)

A total of 45 OAs were enrolled in this study, and there were no dropouts. No significant differences between trial sites were found for age, sex, or years of education (Table 1). However, the trial sites differed significantly in MMSE scores (*F*_2,42_=6.4; *P*=.004; Table 1). Tukey post hoc analysis revealed a significant difference between Switzerland and Cyprus (*P*=.006) and between Italy and Cyprus (*P*=.02). Furthermore, participants from Italy had cognitive (5/15, 33%), neurological (1/15, 7%), orthopedic (10/15, 67%), and cardiac (3/15, 20%) disorders, whereas participants from Switzerland and Cyprus did not have any diagnosed diseases.

**Table 1:** Demographics of older adults (N=45).

		Switzerland (n=15)	Cyprus (n=15)	Italy (n=15)	Total	Range		*P* value	
Age (years), mean (SD)		70.9 (6.4)	67.7 (7.2)	74.6 (9.0)	71.0 (7.9)	59-88		.06	
**Sex, n (%)**							N/A^a^		.15
	Female	8 (53)	4 (27)	9 (60)	21 (47)				
	Male	7 (47)	11 (73)	6 (40)	24 (53)				
MMSE^b^ score, mean (SD)		29.1 (1.0)	27.0 (1.8)	28.9 (2.1)	28.4 (1.9)	23-30		.004	
Years of education, mean (SD)		14.8 (3.0)	15.1 (4.1)	13.6 (4.1)	14.5 (3.8)	5-22		.52	

^a^N/A: not applicable.

^b^MMSE: Mini-Mental State Examination.

#### System Usability (OAs)

The overall SUS score was 68.1 (SD 18.8; n=45) and fell below the predefined 70-point score considered acceptable. When considering the individual countries, the scores differed, revealing acceptable usability in Switzerland (mean 81.5, SD 13.0), borderline acceptable usability in Cyprus (mean 69.3, SD 15.2), and unacceptable usability in Italy (mean 53.5, SD 17.0).

#### Acceptance (OAs)

Table 2 presents the results of the acceptance scores based on the 6 subcategories of the UTAUT (each item was scored on a 5-point Likert scale, 1=strongly disagree; 5=strongly agree). The total mean score (78.9, SD 13.5 out of 100; 78.9% of the total score) indicates high acceptance of the COCARE system among the older participants. Across all 6 categories, the scores were similarly high, with perceived usefulness obtaining the highest score and, thus, demonstrating the highest level of acceptance. When comparing the 3 trial sites, participants from Switzerland exhibited the highest acceptance of the COCARE system, whereas participants from Italy gave lower scores and demonstrated high SDs.

**Table 2:** Acceptance of the Senso Flex based on the Unified Theory of Acceptance and Use of Technology (older adults)^a^.

	Switzerland, mean (SD)	Cyprus, mean (SD)	Italy, mean (SD)	Total, mean (SD)
Perceived ease of use	4.7 (0.5)	3.9 (0.4)	3.6 (0.6)	4.1 (0.7)
Perceived usefulness	4.6 (0.5)	4.2 (0.4)	3.5 (0.8)	4.1 (0.7)
Social influence	3.0 (1.2)	4.1 (0.6)	2.6 (1.1)	3.2 (1.2)
Behavioral control	4.6 (0.4)	4.1 (0.4)	3.4 (0.8)	4.0 (0.8)
Attitude toward use	4.7 (0.5)	4.2 (0.4)	3.3 (0.9)	4.1 (0.9)
Intention to use	4.2 (0.8)	3.4 (0.6)	3.2 (0.9)	3.8 (0.9)
Total score (out of 100)	88.4 (7.9)	81.5 (6.2)	66.9 (14.5)	78.9 (13.5)

^a^Answers were given on a 5-point Likert scale (1=*strongly disagree*; 5=*strongly agree*).

#### Enjoyment (OAs)

Overall, participants from all 3 trial sites rated the enjoyment of playing the exergames with a mean score of 73.3 (SD 12.7) out of 100 points (range 34-96). Across the sites, participants in Switzerland reported the highest enjoyment (mean 82.8, SD 8.7), followed by Cyprus (mean 72.8, SD 8.4), whereas participants from Italy expressed the lowest average enjoyment (mean 63.5, SD 13.1).

#### Safety (OAs)

Most older participants (38/45, 84%) indicated no fear of falling while playing the exergames on the Senso Flex. In terms of safety measures, most participants reported no pain (38/45, 84%) or dizziness (41/45, 91%). Although some participants experienced moments of struggling to maintain balance during the assessments or while playing the exergames, no falls occurred, and the handrail or other forms of lateral support sufficiently satisfied the participants’ desire for safety.

#### Perceived Usability of Single Components of the COCARE System (OAs)

The following results are based on the self-constructed questionnaire addressing various usability-related topics for each component of the COCARE system.

##### Assessment System

**Figure 6:**
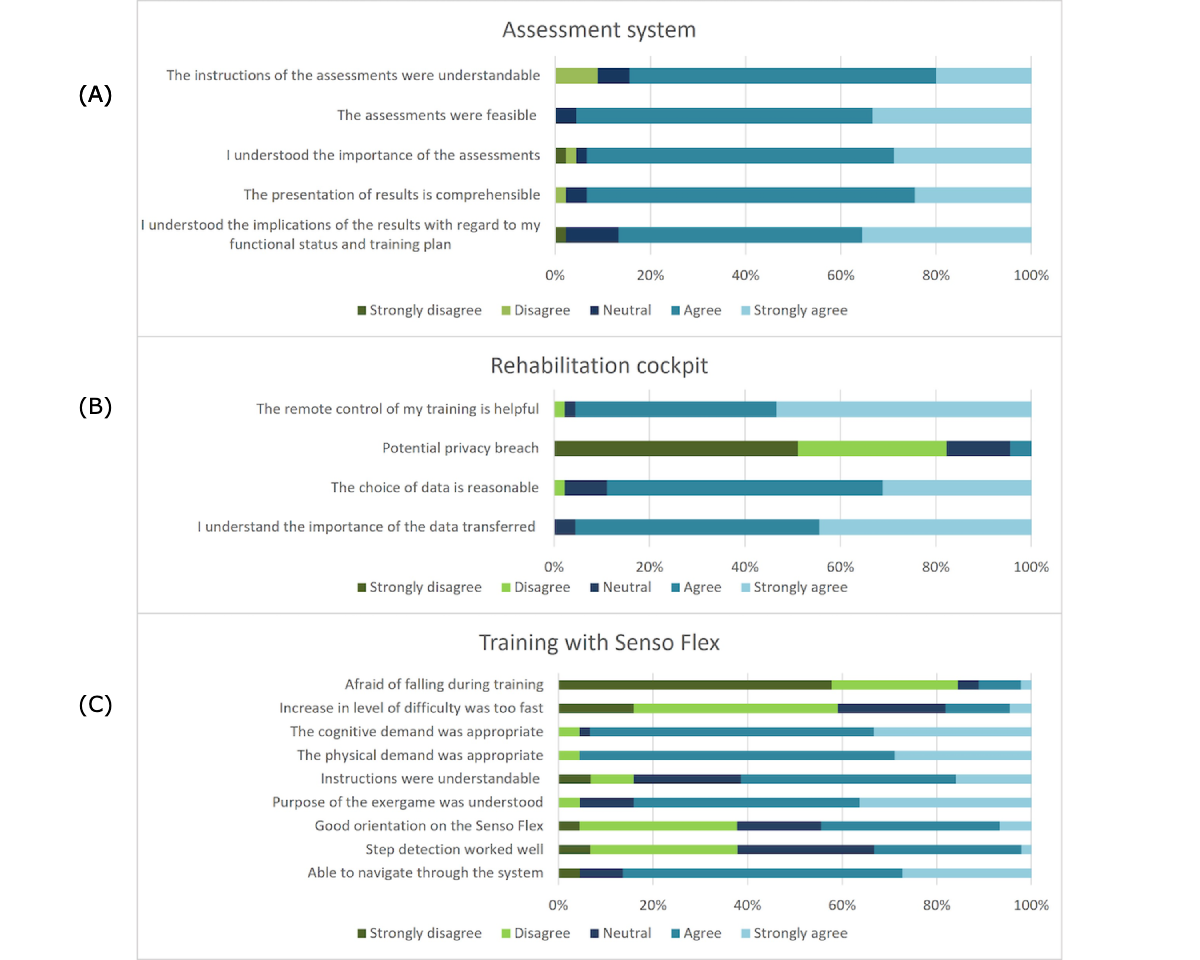
Usability of (A) the assessment system, (B) the rehabilitation cockpit, and (C) the Senso Flex evaluated by older adults.

The perceived usability of the assessments and the assessment report was evaluated very positively, with only 4% (2/45; question 2) to 16% (7/45; question 1) of neutral or negative ratings (Figure 6).

##### Rehabilitation Cockpit

The rehabilitation cockpit also received positive ratings (Figure 6). When asked if there were any dislikes about the system, 89% (40/45) of the participants responded with a “no.” Consequently, the vast majority of participants (43/45, 96%) could envision using the telerehabilitation system as a supplement to their regular physiotherapy.

As the rehabilitation cockpit is also intended to serve as a communication tool for HPs to provide training guidance, the concept of such a communication tool was explained to the participants, and they were further asked about their preferences regarding such a communication system. Among the participants, 58% (26/45) expressed a preference for receiving messages directly on the system, whereas others preferred to communicate via telephone (12/45, 27%), mail (5/45, 11%), or video call (18/45, 40%; multiple answers were possible). Most would like to communicate with HPs once a week (19/45, 42%) or every 2 weeks (10/45, 22%), and only 16% (7/45) would prefer more frequent contact. Concerning messages transmitted through the system, most participants (34/45, 76%) found it important to receive training recommendations, 40% (18/45) expressed interest in also receiving training motivations, and 33% (15/45) expressed interest in receiving training reminders. According to most participants (31/45, 69%), these messages should ideally be sent after training, whereas 49% (22/45) of participants would like to receive messages right before, and only 33% (15/45) during training (multiple answers were possible).

##### Senso Flex and Exergames

The Senso Flex obtained mixed evaluations. Most participants (37/45, 82%) did not perceive any of the setup steps as difficult. Only a small number of participants experienced difficulties when unrolling the mat (1/45, 2%), connecting the mat to the computer (3/45, 7%), turning on the mat (2/45, 4%), turning on the computer (2/45, 4%), and when starting the games (6/45, 13%). On average, the older participants did not report any problems with navigation, understood the purpose of the exergames, and expressed satisfaction with both the physical and cognitive demands posed by the exergames (Figure 6). However, they criticized the step detection sensitivity and experienced some orientation problems, such as difficulties staying in the center of the mat (which is required for correct step recognition). Moreover, a few participants (8/45, 18%) found the software-induced increase in game difficulty level to be too fast. Nevertheless, most participants (38/45, 84%) did not express fear of falling during training.

#### Summary of the Usability Protocol (OAs)

Regarding the Senso, some participants (10/45, 22%) had difficulty finding the correct step length when stepping backward, which, in some cases, resulted in momentary balance issues in the form of short swaying, but no falls occurred. Furthermore, for many participants, the investigators noted good orientation (16/45, 36%), and good body control and balance (20/45, 44%) on the Senso.

The assessment system, especially the option to start each assessment with a warm-up, was praised by some participants, but although the assessment instructions were generally well understood, some participants wished for an additional graphic preview to visualize them, especially for levels 3 and 4 of the Stroop Tests. The participants’ overall view of the assessment report was very positive—particularly for its good comprehensibility and the perceived usefulness of the training recommendations.

Regarding the Senso Flex, most participants did not encounter difficulties during setup apart from minor problems with the correct alignment of the mat. However, a common criticism was related to the low sensitivity of step detection and limited markings of the center area of the mat, which depicts the starting position.

Overall, the exergames were praised primarily for their enjoyment factor, resulting in increased motivation. However, some participants found the game *Simon* challenging to understand as the presentation of stimuli was too fast. In addition, a few participants expressed a desire for more visual input or attractions within the games.

Finally, concerning the rehabilitation cockpit, most participants found it useful and interesting and liked the general idea. Only some participants expressed a wish for a chat section or, preferably, even a video call feature for real-time supervision or explanations. Furthermore, one participant suggested a social platform or community so that patients could also interact with each other.

Multimedia Appendix 2 provides a more detailed overview of the participants’ thoughts along with the investigators’ observations.

#### Secondary Outcomes (OAs)

##### Performance Parameter (Games and Assessments)

Multimedia Appendix 3 demonstrates that, in total, participants from Switzerland performed the best in both games and assessments, followed by participants from Cyprus.

##### Training Motivation

Table 3 shows the OAs’ overall training motivation as well as the results of all subscores on the BREQ-3. Overall training motivation was highest among participants in Switzerland, followed by those in Cyprus. Looking at the subcategories, participants showed high identified and intrinsic regulation, whereas amotivation and external regulation were low.

**Table 3:** Behavioral Regulation in Exercise Questionnaire–3 (BREQ-3) subcategories and total scores (per site and in total; older adults)^a^.

	Switzerland, mean (SD)	Cyprus, mean (SD)	Italy, mean (SD)	Total, mean (SD)
Amotivation	0.0 (0.0)	0.3 (0.5)	0.9 (1.1)	0.4 (0.8)
External regulation	0.1 (0.2)	0.5 (1.0)	1.1 (1.0)	0.6 (0.9)
Introjected regulation	1.5 (0.9)	2.3 (1.1)	2.4 (1.1)	2.1 (1.1)
Identified regulation	3.4 (0.4)	3.4 (0.5)	3.0 (0.9)	3.3 (0.7)
Integrated regulation	3.3 (0.7)	2.9 (1.2)	2.5 (1.4)	2.9 (1.1)
Intrinsic regulation	3.6 (0.4)	3.2 (0.9)	2.8 (1.1)	3.2 (0.9)
BREQ-3 total score (RAI^b^)	19.2 (1.9)	14.8 (6.5)	9.0 (8.7)	14.3 (7.5)

^a^Answers were given on a 5-point Likert scale (0=not true for me; 4=very true for me).

^b^RAI: relative autonomy index.

#### Correlation Between Usability and Secondary Outcomes (OAs)

As shown in Table 4, the performance in all games and assessments exhibited significant correlations with most parameters of usability, enjoyment, and acceptance. Thereby, it had the highest number of significant correlations (medium and large) with the SUS (Spearman ρ=0.35 and *P*=.02 to ρ=0.52 and *P*<.001).

Regarding training motivation, the BREQ-3 showed significant correlations with all usability and acceptance measures except for the UTAUT subcategory *social influence* and large significant correlations with enjoyment (Spearman ρ=0.58; *P*<.01) and the subcategory *attitude* of the UTAUT (Spearman ρ=0.56; *P*<.01).

Looking at the associations of age, we found that age had moderately significant correlations with the SUS (Spearman ρ=−0.35; *P*=.02); the UTAUT total score (ρ=−0.35; *P*=.02); and subscores of acceptance, specifically *attitude toward use* (Spearman ρ=−0.36; *P*=.01) and *intention to use* (Spearman ρ=−0.30; *P*=.04). However, no significant correlations with enjoyment, *perceived ease of use*, and *perceived usefulness* were detected.

Concerning sex and years of education, no significant correlations with any usability measure were found.

**Table 4:** Spearman rank correlations between usability measures and secondary outcomes (older adults).

			SUS^a^		EEQ^b^		Acceptance (UTAUT^c^)												
							Perceived ease of use		Perceived usefulness		Social influence		Behavioral control		Attitude		Intention to use		UTAUT total score
**Age**																			
	r_s_^d^	−0.35^e^		−0.16		−0.18		−0.28		−0.21		−0.24		−0.36^e^		−0.30^e^		−0.35^e^	
	*P* value	.02		.32		.23		.07		.18		.11		.01		.04		.02	
**Sex**																			
	r_s_	0.02		−0.13		−0.12		−0.03		0.17		−0.12		−0.05		−0.08		−0.07	
	*P* value	.88		.39		.44		.85		.26		.43		.72		.63		.64	
**Years of education**																			
	r_s_	0.15		0.06		0.08		0.10		0.24		0.14		0.11		0.08		0.15	
	*P* value	.34		.72		.61		.51		.11		.37		.48		.62		.34	
**BREQ-3^f^ total score (RAI^g^)**																			
	r_s_	0.49^e^		0.58^e^		0.50^e^		0.48^e^		0.03		0.48^e^		0.56^e^		0.50^e^		0.51^e^	
	*P* value	<.001		<.001		<.001		<.001		.84		<.001		<.001		<.001		<.001	
**Targets points**																			
	r_s_	0.35^e^		0.27^e^		0.46^e^		0.22		−0.09		0.31^e^		0.32^e^		0.29^e^		0.36^e^	
	*P* value	.02		.07		<.001		.14		.57		.04		.03		.05		.02	
**Tetris points**																			
	r_s_	0.44^e^		0.28^e^		0.45^e^		0.34^e^		−0.09		0.39^e^		0.41^e^		0.22		0.38^e^	
	*P* value	<.001		.07		<.001		.02		.55		.01		<.001		.14		.01	
**Rocket average speed**																			
	r_s_	0.28		0.06		0.10		0.26		0.31^e^		0.15		0.25		0.08		0.21	
	*P* value	.07		.68		.50		.09		.04		.32		.10		.59		.18	
**Evolve points**																			
	r_s_	0.36^e^		0.14		0.26		0.36^e^		−0.05		0.17		0.28		0.19		0.28	
	*P* value	.02		.37		.08		.02		.72		.27		.06		.21		.06	
**Simon maximum sequence length**																			
	r_s_	0.36^e^		0.49^e^		0.45^e^		0.42^e^		−0.20		0.34^e^		0.38^e^		0.28		0.37^e^	
	*P* value	.01		<.001		<.001		<.001		.18		.02		.01		.06		.01	
**Stroop level 1 average reaction time**																			
	r_s_	−0.51^e^		−0.52^e^		−0.56^e^		−0.46^e^		0.11		−0.54^e^		−0.55^e^		−0.34^e^		−0.49^e^	
	*P* value	<.001		<.001		<.001		<.001		.48		<.001		<.001		.02		<.001	
**Stroop level 2 average reaction time**																			
	r_s_	−0.43^e^		−0.31^e^		−0.53^e^		−0.44^e^		0.08		−0.51^e^		−0.53^e^		−0.24		−0.44^e^	
	*P* value	<.001		.04		<.001		<.001		.61		<.001		<.001		.11		<.001	
**Stroop level 3 average reaction time**																			
	r_s_	−0.52^e^		−0.54^e^		−0.53^e^		−0.45^e^		−0.16		−0.49^e^		−0.46^e^		−0.33^e^		−0.46^e^	
	*P* value	<.001		<.001		<.001		<.001		.28		<.001		<.001		.03		<.001	
**Stroop level 4 average reaction time**																			
	r_s_	−0.43^e^		−0.46^e^		−0.44^e^		−0.50^e^		−0.06		−0.50^e^		−0.56^e^		−0.45^e^		−0.53^e^	
	*P* value	<.001		<.001		<.001		<.001		.70		<.001		<.001		<.001		<.001	
**Coordinated stability completeness of the path**																			
	r_s_	0.43^e^		0.35^e^		0.39^e^		0.47^e^		−0.13		0.41^e^		0.50^e^		0.35^e^		0.44^e^	
	*P* value	<.001		.02		.01		<.001		.40		.01		<.001		.02		<.001	

^a^SUS: System Usability Scale.

^b^EEQ: Exergame Enjoyment Questionnaire.

^c^UTAUT: Unified Theory of Acceptance and Use of Technology.

^d^Spearman rank correlation coefficient.

^e^The correlation was significant at a significance level of .05 (2-sided).

^f^BREQ-3: Behavioral Regulation in Exercise Questionnaire–3.

^g^RAI: relative autonomy index.

### Secondary End Users (HPs)

#### Demographics (HPs)

A total of 15 HPs were enrolled in this study, and there were no dropouts. Comparing the demographics of the 3 trial sites, no significant group differences in terms of age, sex, and experience in the health care field and working with OAs were found. In addition, overall, sex distribution was balanced in this study (Table 5).

**Table 5:** Demographics of health care professionals (N=15).

		Switzerland (n=5)	Cyprus (n=5)	Italy (n=5)	Total	*P* value	
Age (years), mean (SD)		28.8 (2.7)	31.4 (5.3)	36.4 (12.5)	32.2 (8.1)	.35	
**Sex, n (%)**							.15
	Female	1 (20)	4 (80)	3 (60)	8 (53)		
	Male	4 (80)	1 (20)	2 (40)	7 (47)		
Number of years in health care, mean (SD)		5.6 (3.4)	6.4 (3.2)	9.8 (6.6)	7.3 (4.7)	.35	
Number of years of work with OAs^a^, mean (SD)		5.4 (3.2)	5.0 (3.5)	9.0 (6.5)	6.5 (4.7)	.36	

^a^OA: older adult.

#### System Usability (HPs)

The overall SUS score for HPs was 70.7 (SD 12.3; n=15), slightly surpassing the predefined acceptable threshold of 70 points. However, looking at site differences, participants from Cyprus and Italy rated the system with mean scores of 65.5 (SD 9.42) and 65.5 (SD 6.47) points, respectively, whereas in Switzerland, this score was significantly higher (mean 81.0, SD 13.99 points).

#### Acceptance (HPs)

Table 6 presents the results of the UTAUT questionnaire measuring acceptance through 6 subcategories (each item rated on a 5-point Likert scale). The total mean score (85.1, SD 8.3 out of 105; 81% of the total score) indicates high acceptance of the COCARE system among the HPs. Notably, all 6 categories received similarly high scores, with attitude toward use and intention to use receiving the highest scores. Although the results were generally similar among all investigation sites, participants from Switzerland awarded the highest acceptance scores overall.

**Table 6:** Acceptance of health care professionals based on the Unified Theory of Acceptance and Use of Technology (UTAUT).

	Switzerland, mean (SD)	Cyprus, mean (SD)	Italy, mean (SD)	Total, mean (SD)
Perceived ease of use	4.0 (0.7)	3.5 (0.4)	3.5 (0.2)	3.7 (0.5)
Perceived usefulness	4.3 (0.7)	4.2 (0.2)	4.2 (0.4)	4.2 (0.4)
Social influence	4.0 (1.0)	3.2 (1.1)	3.8 (0.5)	3.7 (0.9)
Behavioral control	4.4 (0.8)	4.0 (0.4)	3.7 (0.3)	4.0 (0.6)
Attitude toward use	4.6 (0.3)	4.5 (0.2)	4.1 (0.2)	4.4 (0.3)
Intention to use	4.4 (0.9)	4.5 (0.6)	3.9 (0.4)	4.2 (0.7)
Average	4.3 (0.6)	4.0 (0.2)	3.9 (0.2)	4.0 (0.4)
Total UTAUT score (out of 105)	89.6 (12.9)	84.4 (4.8)	81.4 (2.9)	85.1 (8.3)

#### Safety (HPs)

The issue of safety for OAs when training independently using the Senso Flex sparked disagreements among HPs. A total of 33% (5/15) of HPs considered independent use safe, whereas 47% (7/15) remained uncertain and 20% (3/15) even perceived a significant lack of safety. The primary concern raised by HPs was the absence of a handrail, which they felt should be available, especially for OAs with a fear of falling or those with certain medical conditions.

#### Usability of Single Components of the COCARE System (HPs)

##### Assessment System

**Figure 7:**
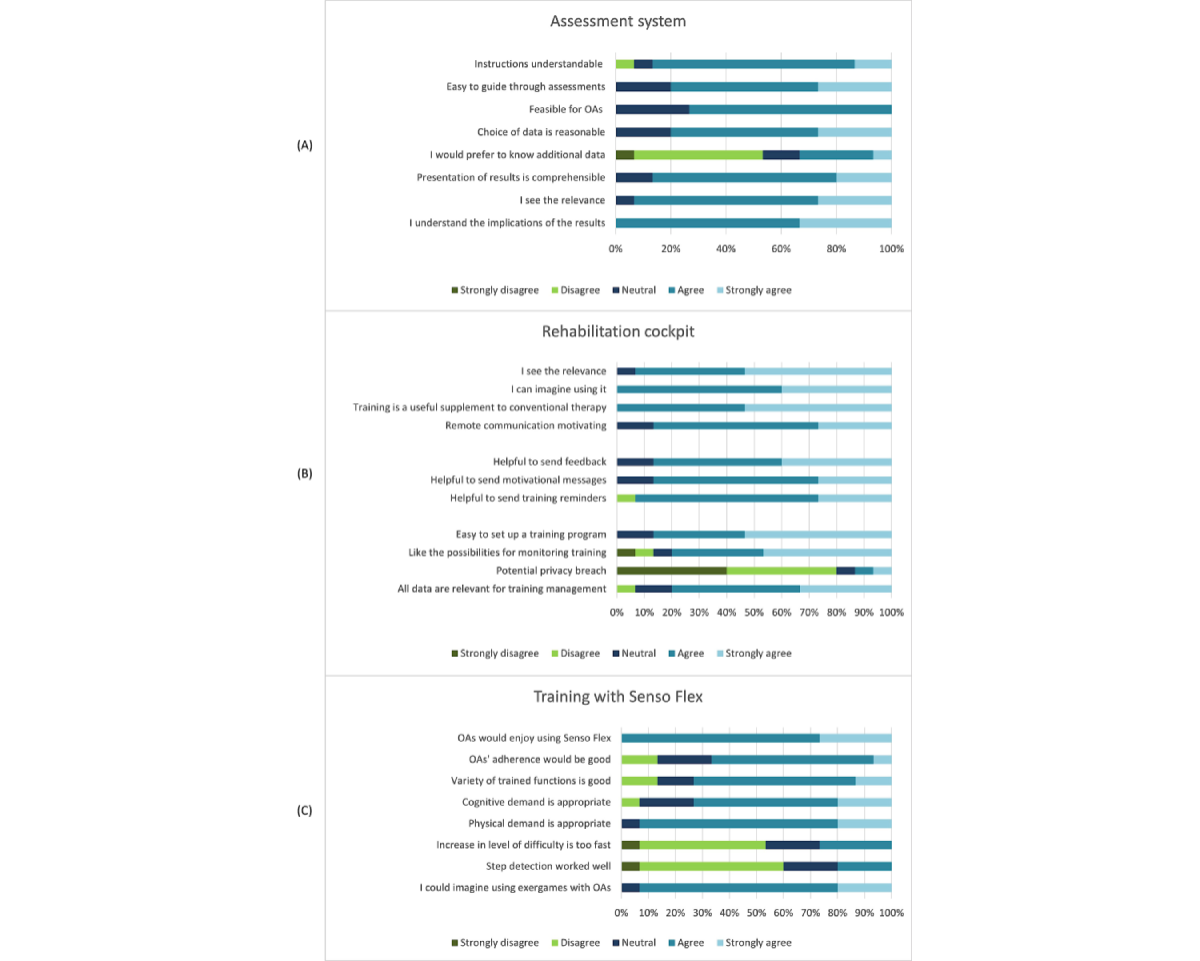
Usability of (A) the assessment system, (B) the rehabilitation cockpit, and (C) the Senso Flex evaluated by health care professionals. OA: older adult.

Overall, HPs provided favorable ratings for the assessment system (Figure 7). Although some HPs (4/15, 27%) remained neutral regarding the feasibility of the assessments, most (14/15, 93%) recognized the relevance of the assessment system and found the instructions, as well as the assessment report, to be comprehensible. Furthermore, all HPs (15/15, 100%) demonstrated an understanding of the implications of the assessment results regarding further training management. However, 33% (5/15) of HPs wished for additional data to be presented in the assessment report, such as body weight distribution, accuracy, and a comparison based on different age groups and sex.

##### Rehabilitation Cockpit

The rehabilitation cockpit received positive evaluations (Figure 7), and accordingly, all participants (15/15, 100%) could envision supervising and managing their patients’ training with its assistance.

In terms of future ways of communicating with their patients, most HPs would find it useful to send training reminders (14/15, 93%), motivational messages (13/15, 87%), and training feedback. A total of 87% (13/15) of HPs regarded messages on the system as a favorable option, and more than half (8/15, 53%) of HPs would like to have video calls as well. In contrast, communicating via telephone was perceived as less appealing, and similarly, only 7% (1/15) of the participants considered sending emails a suitable means of communication.

##### Senso Flex and Exergames

Questions related to the Senso Flex primarily concerned its setup and navigation through the games. A total of 20% (3/15) of the participants would not expect any difficulties with any setup step. However, most HPs (9/15, 60%) found it challenging to connect the Senso Flex to the computer, unroll the mat (1/15, 7%), turn on the mat (5/15, 33%), turn on the computer (5/15, 33%), and start the games (6/15, 40%). Consequently, most HPs (9/15, 60%) believed that external support or a caregiver would be necessary.

Regarding an exergame-based training on the Senso Flex, opinions were positive (Figure 7). Most HPs felt that the physical (14/15, 93%) and cognitive demand (11/15, 73%), the variety of trained functions (11/15, 73%), and the increase in the level of difficulty (8/15, 53%) were appropriate. In addition, all HPs (15/15, 100%) expected OAs to enjoy using the Senso Flex. Nevertheless, not all HPs believed that OAs would adhere to such a training program. In addition, a major problem for HPs was a low step detection sensitivity. Nonetheless, most HPs (14/15, 93%) could envision integrating the exergames into their training plans.

#### Summary of the Usability Protocol (HPs)

HPs did not share many opinions or suggestions for further development of the Senso but focused more on the other components of the COCARE system. Regarding the assessment system, they found the UI and navigation easy and user-friendly, praising the warm-up feature as well as the possibility of repeating the warm-up as often as needed. However, they were more critical of the Stroop Test, questioning its feasibility and comprehensibility. In addition, some HPs felt that the Coordinated Stability Test could be too demanding for OAs.

Regarding the assessment report, HPs appreciated the general structure and training recommendations; they only wished for simpler explanations of specific terms such as *executive function* and *percentile*. Some HPs were also concerned that the classification in percentiles might have a demotivating effect on patients.

HPs’ criticisms of the Senso Flex aligned with the OAs’ requirements. For instance, the low step detection sensitivity of the mat and lack of demarcation of the center area were common concerns shared by HPs and OAs. In this regard, it was suggested to create an embossed border or tactile texture separating the center area from the outer fields. Finally, a few HPs expressed concerns about the risk of falling, which is why they proposed providing lateral support through chairs or walkers.

The exergames were viewed very positively by HPs, who found them to be a good challenge, good exercise, and enjoyable. In addition, most HPs described the instructions as understandable and intuitive. Only some suggested the inclusion of pictures or animations to illustrate the instructions. When evaluating the games separately, *Targets* and *Evolve* received very positive feedback, whereas *Simon* was confusing for some HPs because of the fast presentation of stimuli, and walking on the spot—as required in *Rocket*—was criticized a few times for being an unnatural type of walking without a clear aim or reward.

Finally, when commenting on the rehabilitation cockpit, HPs particularly praised its overall usefulness and the clear overview of training progress. Furthermore, they found the general UI and especially the setting possibilities to be simple and intuitive. Nevertheless, a few HPs rated other therapists’ and older patients’ acceptance of remote therapy and constant monitoring as low. Moreover, HPs suggested some improvements regarding future communication possibilities, namely, the integration of a video call feature, a chat section, or a real-time audio-video connection.

In Multimedia Appendix 4, a more detailed overview of the HPs’ thoughts can be found.

## Discussion

### Overview

This study aimed to investigate the usability of the newly developed exergame-based COCARE system for telerehabilitation in OAs. Usability was assessed quantitatively and qualitatively, and valuable insights into the perspectives of OAs and HPs regarding the COCARE system was gained. Overall usability, enjoyment, acceptance, and safety ratings were acceptable. The analysis revealed that some parts of the system need improvement—especially regarding comprehensibility of assessments and game instructions and hardware features. Almost all secondary outcomes showed manifold correlations with the usability outcomes. Each of these outcomes will be discussed in the following sections.

### Overall Usability

The overall usability of the system, quantitatively assessed using the SUS, was rated with a mean score of 68.1 (SD 18.8; OAs) and 70.7 (SD 12.3; HPs) points. A score of 70 points has been defined as “fully acceptable” [18,39,55], whereas a score of <50 points has been interpreted as truly nonacceptable [39]. On the basis of these definitions, the COCARE system’s usability can be considered acceptable.

It is worth noting that usability scores from previous studies on similar exergame systems vary, with some studies showing slightly higher [18,56,57] or even significantly higher SUS scores [14,57]. However, in all these studies except one [57], usability was assessed after 10 to 24 training sessions, whereas in this study, the COCARE system was evaluated after only 1 exergame session.

In contrast, Thalmann et al [55] investigated a similar home-based multicomponent exergame training system for OAs that received lower SUS scores compared with the COCARE system. This difference could potentially be explained by the inclusion of participants with mobility limitations and a higher mean age (80.5, SD 4.9 y) in the study by Thalmann et al [55]. Looking at other previous studies [57-60], the latter factor is especially likely to result in a lower SUS score. For instance, Baschung Pfister et al [57] conducted a usability study on an interactive tablet-based exercise application for independent home-based training, which was, similar to the COCARE system, developed by researchers from ETH, University Hospital Zürich, and Dividat AG. Participants in that study were healthy younger adults with a mean age of 38 (SD 9) and OAs with a mean age of 57 (SD 10), and the application indeed obtained higher SUS scores in the younger participants. This study’s results also support the assumption that age significantly influences usability as significant correlations were found between age and several usability measures, including SUS score, attitude toward use, intention to use, and UTAUT total score.

### Usability of the Single Components of the COCARE System

#### Assessment System

The assessment system received positive ratings in the questionnaire from both OAs and HPs. However, when asked to think aloud, participants indicated difficulties in understanding the instructions provided by the system, suggesting the integration of videos or pictures to visualize the instructions. These evaluations indicate the importance of a well-structured system, starting with easy assessments before moving on—and only if necessary—to more advanced assessments.

#### Senso Flex

Concerning the Senso Flex, a crucial aspect for OAs is its setup demands. Observations made by the investigators and the perceived level of difficulty reported by the OAs indicated that, on average, the older participants performed very well in setting up the system. However, it became evident that HPs significantly underestimated OAs’ ability to properly set up and operate the Senso Flex. Similarly, HPs expressed concerns about the risk of falls when OAs train independently using the Senso Flex—a concern not shared by most OAs themselves. Both discrepancies were previously observed in the first study (focus group study) of the COCARE project [27] and are consistent with findings from earlier investigations [61]. Presumably, these discrepancies are rooted in ageism existing even among HPs [62], possibly because of their experiences with older patients who have severe mobility limitations. However, it is noteworthy that 67% (30/45) of older participants (all from Switzerland and Cyprus) in this study were physically and cognitively healthy, not fitting the aforementioned stereotype.

Nevertheless, participants in Switzerland and Italy repeatedly reported sensitivity issues with the Senso Flex, resulting in incorrect step detection. In addition, participants from both groups at all sites criticized specific hardware and software issues, namely, missing handrails, problems with internet connection, and orientation difficulties on the mat because of insufficient visual or tactile demarcations of the fields. All these issues, along with technological malfunctions, likely had a substantial impact on the deduction of SUS scores. Consequently, in the further evolution of the COCARE system, resolving these software and hardware problems is crucial to enhance its usability and acceptance.

#### Exergames

Despite encountering several difficulties, both OAs and HPs expressed overall satisfaction with the exergames as they recognized the potential physical and cognitive benefits of the exergame training and awarded high exergame enjoyment scores. This is in line with previous literature, which indicates that exergames are accepted by and usable for healthy OAs [15,25,29,56,63], with exergame enjoyment playing a significant role in their acceptance [64]. However, 2 specific games (*Simon* and *Rocket*) received criticism for their high level of difficulty, leading to confusion among OAs. As a result, providing good guidance and improved instructions emerged as critical factors not only for these games but also for enhancing the overall usability of other games in the system.

#### Rehabilitation Cockpit and Telerehabilitation

The rehabilitation cockpit, serving as a tool for telerehabilitation, garnered positive feedback from both OAs and HPs. Participants found it highly useful and interesting for patients as well as for HPs. These observations are in accordance with previous research, which highlighted that OAs recognize the value of mobile health—a form of telerehabilitation. Specifically, mobile health and telemedicine have been found to be effective, for instance, in treating noncommunicable diseases [65] and have been shown to be feasible, enhancing communication, social interaction, and access to information; providing a feeling of security; and facilitating independent living [66,67]. These factors may explain the findings of previous studies indicating that remote support can increase exercise adherence [68]. Similarly, participants in this study expressed interest in future communication possibilities. This aligns with previous research showing that social interaction and individual feedback play crucial roles in the acceptance of telerehabilitation [27] and that individual feedback potentially increases the motivation to learn new skills [69,70] as long as it is evaluative and not comparative [70].

However, previous studies have also identified common barriers to adherence and effectiveness of telerehabilitation. These include, for instance, technological literacy, internet access, usability, education, social support, perceived need, and costs [66,67,71]. These factors might explain why, despite the positive feedback from OAs, a few HPs remained critical of OAs’ acceptance of the rehabilitation cockpit and telerehabilitation in general. Consequently, it is essential to educate both OAs and HPs on the benefits of telerehabilitation and promote technological literacy, particularly among OAs.

Surprisingly, and contrary to other studies [72], most HPs and OAs did not express concerns about privacy and confidentiality. This aligns with the agreement on the choice of data transferred to the HPs and indicates a level of comfort with the system’s data-handling protocols.

### Enjoyment

The average EEQ scores point to a satisfying enjoyment of the exergames—a result supported by the qualitative analyses of satisfaction with the exergames. Manser et al [73] used similar Dividat exergames to investigate the validity of a German translation of the EEQ in OAs and found similar enjoyment scores. Furthermore, these findings are consistent with those of previous studies by Altorfer et al [14] and Jäggi et al [13], who tested the feasibility of Dividat exergames played on the Senso in different rehabilitation clinics and geriatric inpatient groups, reporting high mean enjoyment levels of 4.78 (SD 0.52) and 4.51 (SD 0.73), respectively, on a 5-point Likert scale. Further studies involving other exergame devices have yielded similar results. For instance, Graves et al [74] demonstrated that Wii Fit tasks were more enjoyable than sedentary video game play or treadmill training for OAs. In general, enjoyment can be considered a crucial advantage of exergames as it has exhibited strong associations with OAs’ intrinsic training motivation [75], potentially contributing to the increased adherence in exergame training sessions observed in previous studies [14,64]. Consequently, enjoyment is likely one of the most important aspects of the usability of exergames.

Drawing on this assumption, Sweetser and Wyeth [76] developed and validated the GameFlow model, a model of player enjoyment in games describing the motivators that enhance a user’s interest in playing (computer) games. This model identifies eight core elements crucial for game enjoyment, most of which are also included in the EEQ: (1) the game should require some concentration and (2) be challenging but (3) match the player’s skill level, (4) the player should have some control, (5) the game should have clear goals, (6) appropriate feedback should be given, (7) there should be immersion in the game, and (8) social interaction should be possible. Considering this model along with the results of the EEQ, the high enjoyment scores for the Senso and Senso Flex can be well explained as participants in this study found that most core requirements were met. Thus, according to many participants, the games were challenging and required concentration, most matched the players’ skill level, and immediate feedback was provided. However, these core elements and the participants’ feedback also indicate areas for improvement in the COCARE system to enhance enjoyment. Some games must be adapted to the OAs’ skills (especially *Simon*), and others should have clearer goals (*Rocket*). In addition, the integration of possibilities for social interactions in the games should be considered, as suggested in previous studies [77] and in the focus groups within the COCARE project [27].

### Acceptance

The overall acceptance ratings based on the UTAUT were high among both OAs and HPs. These findings align with those of Baschung Pfister et al [57], who investigated the acceptance of an interactive tablet-based exercise application sharing many characteristics with the COCARE system and obtained comparable results. Despite using the TAM as a measure for acceptance and having slightly younger participants (mean age of 57, SD 10 y), their findings support the assumption that remotely managed training using ICTs is generally accepted by older patients, as is the use of technologies for exergaming.

Analyzing the UTAUT subcategories, *perceived usefulness* followed by *perceived ease of use* received the highest scores from OAs, whereas HPs’ acceptance of the COCARE system was mainly driven by their *intention to use* and *attitude toward use* followed by *perceived usefulness*. This difference between OAs and HPs is well in line with previous studies [72]. Furthermore, the positive evaluation of *perceived ease of use* confirms the results of the SUS, indicating that, apart from the aforementioned software and hardware issues, the system was generally considered usable. The high scores on the subcategories *attitude toward use* and *intention to use* by HPs demonstrate their willingness to indeed integrate the system into their therapy.

Surprisingly, *social influence*, for instance, recommendations by caregivers or colleagues, seemed to play a minor role for OAs and HPs in the acceptance of the system, which deviates from the findings of previous studies [41,78-81]. The extent of social influence may be dependent on cognitive status, with individuals experiencing cognitive impairment typically exhibiting greater reliance on others. The combination of significantly lower MMSE scores and higher social influence measures in Cyprus compared with the other study sites supports such an association. However, the lowest *social influence* score detected in Italy might be mainly attributable to the overall lower acceptance scores compared with the other 2 study sites. A possible explanation for this is that, from a cognitive perspective, personal experience usually overrides external opinions or advice.

### Safety

Despite momentary balance issues in a few participants, most OAs did not report fear of falling when using the Senso Flex, and only a small number of participants experienced pain or dizziness while playing the exergames, with no adverse events. This aligns with a review conducted by Valenzuela et al [64], who analyzed adverse events related to technology-based exercise programs in OAs and found only 1 study reporting minor adverse events. Similarly, no adverse events have been reported in other exergame intervention studies conducted since then [13-15,20,75]. Although a definitive safety analysis requires examination over an extended training period, including autonomous home use of the system, the results obtained in this study based on a single supervised session are promising regarding the safety of the device.

### Influencing Factors

The secondary aim of this study was to analyze possible correlations among potential influencing factors, namely, age, sex, years of education, training motivation, game and assessment performance, and measures of usability. Except for sex and years of education, many significant correlations were found, with the SUS exhibiting the highest number of associations with all secondary outcome measures—most likely because of its comprehensive assessment of overall usability covering all other measures of usability. Concerning sex and years of education, the results of previous studies are controversial [78], with some indeed reporting an impact of these sociodemographic factors on attitudes toward and use of technologies in OAs [41,60,82] and others not [78]. However, it is worth noting that these studies investigated the acceptance of general computerized [60] or tracking systems [41,82], whereas this study investigated a specific technological system designed to be user-friendly and enjoyable, which may explain the limited role of sex and years of education.

Regarding training motivation and performance measures, the direction of the effects must be further evaluated. Possibly, a highly usable device fosters higher motivation and better performance, but conversely, motivated individuals or those performing well in the games may perceive the system as more usable than others.

Moreover, it must be considered that the differences in performance, acceptance, and usability ratings were primarily attributed to the participants’ country affiliation, with participants from Switzerland showing the best performance and giving the highest usability and acceptance ratings, whereas those in Italy generally exhibited much lower values in all outcome measures. Possible explanations for this disparity include the fact that participants recruited in Italy had a higher, though not statistically relevant, mean age and had various disorders, in contrast to participants enrolled in Switzerland and Cyprus, who were physically and mentally healthy. In addition, cultural and family structure differences may have played a role as people in Italy and Cyprus tend to live in larger families with stronger bonds compared with Northern European countries and their “contemporary Western lifestyle” [83]. Shirahada et al [79] suggested that, in individualist countries—to which Switzerland most likely belongs—with family members living further apart, OAs are more dependent on mobile communication. Similarly, according to Michailidou et al [83], OAs in Cyprus prefer offline settings for any type of support as this support is mainly provided by family members. Thus, it can be assumed that a less frequent ICT use in countries such as Italy and Cyprus is associated with a lower technology acceptance. This assumption is supported by previous research showing that Swedish OAs were more frequent users of technologies and had more positive attitudes toward ICTs compared with OAs in Italy [60].

### Strength and Limitations

The strength of this study is that (contrary to previous usability studies) it not only directly compares the opinions and demands of HPs and OAs but also provides valuable insights into country- and culture-related differences within both participant groups. Moreover, this study points to future pathways for developing feasible, user-friendly, and enjoyable exergame systems tailored for home settings.

A major limitation of this study is that the system could only be tried out once, mainly because of COVID-19 restrictions. Nevertheless, the participants’ feedback indicated that they were able to immerse themselves in the exergame experience and that their expressed opinions were not solely based on their one-time gaming session but also took potential long-term use into account. Future feasibility trials will provide deeper insights into the usability and acceptance of the COCARE system when used over a longer period.

### Conclusions

This study revealed some differences between OAs and HPs in terms of their perception of usability and acceptance of the COCARE system. OAs demonstrated higher acceptance of the system and better performance on the Senso and Senso Flex and found the setup of the Senso Flex to be easier than expected by most HPs. Furthermore, OAs were less concerned about the potential risk of falls compared with HPs.

Disparities also emerged among the study sites concerning all usability and acceptance ratings, possibly stemming from cultural differences in the significance and proximity of family and the resulting motivation to integrate ICTs into everyday life.

Several important requirements were identified by both OAs and HPs, which should be considered in further development efforts to enhance the usability of these and other technology-based telerehabilitation training systems. These include improvements in mat sensitivity, markings on the mat for a better orientation, stable internet connection, simplification of the instructions and results presentation for some assessments, adaptation of some games and their instructions (eg, video instructions) to be more usable and enjoyable for OAs (especially *Simon* and *Rocket*), and integration of social interaction possibilities. Nevertheless, the overall high scores for usability and acceptance indicate that many of these negative aspects listed in the usability protocol do not significantly impair the usability, acceptance, and enjoyment of the COCARE system, warranting further longitudinal studies spanning weeks of training or exergaming. Thus, subsequent adaptations should be followed by feasibility and effectiveness testing, including safety confirmation, in larger field trials.

## Appendix

Multimedia Appendix 1 STROBE (Strengthening the Reporting of Observational Studies in Epidemiology) checklist.

Multimedia Appendix 2 Usability protocol: comments, questions, and observations by older adults and the investigator.

Multimedia Appendix 3 Performance of older adults in games and assessments.

Multimedia Appendix 4 Usability protocol: comments, questions, and observations by health care professionals and the investigator.

## Abbreviations

BREQ-3: Behavioral Regulation in Exercise Questionnaire–3
COCARE: Continuum-of-Care
EEQ: Exergame Enjoyment Questionnaire
HP: health care professional
ICT: information and communications technology
MMSE: Mini-Mental State Examination
OA: older adult
STROBE: Strengthening the Reporting of Observational Studies in Epidemiology
SUS: System Usability Scale
TAM: Technology Acceptance Model
UCD: user-centered design
UI: user interface
UTAUT: Unified Theory of Acceptance and Use of Technology

## Data Availability

The raw data supporting the conclusions of this study will be made available by the authors without undue reservation to any qualified researcher upon request.
