# Supplementary material for: A Newly Developed Exergame-Based Telerehabilitation System for Older Adults: Usability and Technology Acceptance Study
Source: JMIR Hum Factors. 2023 Dec 7;10:e48845. doi: 10.2196/48845 (PMC10739244; doi:10.2196/48845)
Supplement: Multimedia Appendix 2 [file humanfactors_v10i1e48845_app2.docx]

## **Multimedia Appendix 2: Usability Protocol: comments, questions, and observations by OAs and the investigator**

| **Older adults** | | |
| --- | --- | --- |
| **Category** | **Positive aspects** | **Negative aspects, requirements, and suggestions for development** |
| **Senso** | | |
| **Orientation on the Senso** | - Good (n_S_=10) (n_C_=4) (n_I_=2) | - Difficulties with required step length (back step too long, returning to central position is difficult) (n_S_=3) (n_C_=1) (n_I_=6) |
| **Balance and safety** | - Good body control and balance (n_S_=4) (n_C_=8) (n_I_=8) - Secure standing without using the handrail (n_S_=1) (n_C_=8) (n_I_=6) | - Loss of balance/stability difficulties (especially when stepping backwards) (n_S_=6) (n_C_=2) (n_I_=1) - Feeling of insecurity in the beginning of the session (n_C_=2) - Usage of the handrail for support during playing of the exergames (n_S_=5) (n_C_=7) (n_I_=4) |
| **Size of the central square** | - Good dimension (n_I_=3) | - Too small (difficult for people with large feet) (n_S_=1) (n_I_=1) |
| **Step detection sensitivity of the Senso** | - High (n_S_=8) | - Too low (n_S_=1) (n_C_=1) |
| **Effort** | - Very concentrated while playing (n_C_=2) and showed good effort (n_C_=4) |  |
| **Assessment System** | | |
| **Stroop** | | |
| **Content** | - Interesting (n_C_=2) - Mostly easy (n_C_=16) (n_I_=1) - Good for the mind (n_C_=2) - *“I like it a lot and it is a very good tool for older adults”* (P_C_305) |  |
| **Instructions** | - Clear (n_I_=5) - Well understood in most levels (especially level 1: (n_S_=14) (n_C_=15) (n_I_=2) and level 2: (n_S_=11) (n_C_=14) (n_I_=3)) - Warm-up phases helpful to fully understand the assessment (n_I_=4) | - Oral Repetition of instructions required in (especially level 3 ((n_S_=13) (n_C_=2) (n_I_=5)) and level 4 ((n_S_=6) (n_I_=5)) - Participant had to read the instructions several times to memorize them (n_C_=3) - At the beginning instructions were well understood, but while performing the participant got confused (n_C_=6) - A graphic preview to visualize the instructions would be useful (n_I_=2), (n_S_=1) |
| **Performance** | - Good in levels 1 (n_S_=4) (n_C_=12) (n_I_=5) and 2 (n_S_=3) (n_C_=13) (n_I_=5) | - Difficult in levels 3 (n_S_=4) (n_C_=3) and 4 (n_S_=4) (n_I_=6) - Unable to perform the assessment independently (n_I_=2) |
| **Coordinated Stability** | | |
| **Content** |  | - Concerns about patients who are less fit, having balance problems or mild cognitive impairment (n_S_=1) (n_I_=1) |
| **Instructions** | - Understood (n_S_=6) | - Not fully understood (n_C_=1) (n_I_=2) |
| **Safety** | - Performed without handrail (n_C_=2) | - Perceived risk of a fall (n_I_=1) - Use of handrail for safety purpose (instead of crossing the arms in front of the chest as required) (n_S_=7) (n_C_=13) - Loss of balance (n_I_=1) |
| **Performance** | - Very good (n=4) (n_I_=5) - Good body control and balance (n_S_=2) (n_C_=8)   (n_I_=3) | - Required movements are challenging, common mistakes: movement only of the upper body (n_S_=4) (n_C_=1) (n_I_=2), lifting or moving the feet (n_S_=1) (n_I_=2), too fast (n_S_=2) (n_C_=1) - Difficult to reach the “edges” (especially when holding the handrail) (n_S_=2) (n_C_=5) - Coordination problems (n_C_=6) (n_I_=2) - Not enough time to finish (n_C_=4) - *“Does velocity or accuracy matter?” (P114)* |
| **Assessment Report** | | |
| **Usefulness** | - Training recommendations useful for HPs and patients (n_S_=10) (n_I_=4) - *“It is good to know about my results and how I performed”* (n_C_=5) - *“It could be useful for recommendations on how to train. I appreciate it when my trainer in the gym often varies the proposed exercises.”* (P_I_501) | - Not useful (n_C_=3) |
| **Comprehensibility** | - Clear and understandable (n_S_=5) (n_C_=3) (n_I_=3) - Explained in a simple manner (n_C_=4) - Reasonable structure (n_S_=1) - The categories are clear and complete (n_I_=2) - *“The results are well explained, and I like the fact, that I could keep a copy of the results*” (n_C_=2) - *“I did not understand what the term “executive functions” means, but after reading the explanation it was clear”* (P_S_105) | - Not completely understandable (n_I_=2) - Unclear terms (“inhibition”, “executive function”) (n=5) (n_I_=2) - Unclear term “percentile” (n_S_=2) (n_I_=5) - Explanations should be simpler (n_S_=1) |
| **Motivation** | - *“The percentile is the most important value”* (P_I_514) | - Should be formulated in a more motivational manner (n_S_=1) - *“It would be nice to have a diagram or a line/graph where you can see how you performed compared to your age group”* (P_S_114) |
| **SensoFlex** | | |
| **Set-up** | - No difficulties (n_S_=12) (n_C_=13) (nI=7) | - Help needed (e.g., due to memory problems) (n_C_=2) (n_I_=1) - Difficulties to unroll the mat due to shoulder or back pain (n_I_=2), to align it (which side must point towards the TV) (n_I_=4), or to bend down to turn the Senso Flex (n_S_=1) - *(“(…) more intuitive to indicate it* [alignment of the mat] *with symbol or word”* (P_I_513);*“useful to draw a pair of feet in the center of the mat to indicate the direction it has to be placed”* (P_I_515)) |
| **Navigation** | - Worked well (n_S_=2) (nI=1) | - Difficulties (e.g., the game was quit by mistake or participant forgot long step forward for game selection) (n_S_=2) (n_C_=2) (n_I_=8) |
| **Orientation** | - No deviations from the center) (n_S_=1) (n_C_=4) - No need to further delimit the areas on the mat (n_I_=2) | - Difficult (due to no marking of the middle plate) (n_S_=9) (n_C_=9) (n_I_=4) - Middle plate should be marked in a different color or be embossed (n_C_=2) |
| **Balance and Safety** | - Well balanced (n_C_=6) (nI=1) - No fatigue or need to stop during playing the exergames (n_I_=2) - *“There is no need to add handrails or protections against falls”* (P_I_501) | - Balance difficulties (n_C_=2) (n_I_=1) - Lateral support required (e.g., chairs) (n_I_=6) |
| **Step detection sensitivity** |  | - Low (n_S_=8) (n_I_=8), frustrating (n_S_=3) |
| **Exergames** | | |
| **Instructions** | - Well understood, especially in case of Targets (n_S_=11) (n_C_=12) (n_I_=5) and Evolve (n_S_=7) (n_C_=11) (n_I_=2) | - Difficulties in understanding, especially in case of Evolve (n_S_=4) (n_C_=8) (n_I_=2) and Simon (n_S_=1) (n_C_=15) (n_I_=4) |
| **Comprehensibility** |  | - Confusion while playing Simon because the presentation of stimuli happens too fast (n_S_=2) (n_C_=15) (n_I_=10) - Aim or logic of some games was unclear (n_I_=2) |
| **Performance** | - Good in Targets (n=2) (n_C_=4), and Rocket (n_C_=12) | - Difficulties in certain movements (e.g., too much movement of the upper body) while playing Evolve (n_I_=1) (n_C_=7) |
| **Enjoyment and motivation** | - Games were liked for fun (n_S_=1) (n_C_=3) (n_I_=2), excitement (n_C_=2), the challenging aspect (n_C_=4) - Participants were motivated (n=1) and into the games (n_C_=3) - Good Pastime (n_I_=1) - *“It makes time pass”* (P_I_504) - *“I like the fact that this game requires full body movement”* (n_C_=2) | - Wish for more visual input/more attractions (n_S_=2): *“It would be nice if the rocket would fly faster so there is a chance to see the space for all participants and not only for the fast participants”* (P_S_112) |
| **Safety** |  | - Rocket: Walking on the spot is an unnatural type of walking (n_I_=6), tiring (n_I_=4) and caused knee pain (n_I_=2); game had to be interrupted due to exhaustion (n_I_=2) |
| **Rehabilitation Cockpit** | | |
| **Comprehensibility** | - Good (n_S_=1) (n_I_=6) |  |
| **Usefulness** | - Useful and interesting (n_S_=8) (n_I_=1) - Loved the recommendations (n_S_=4) - *“Totally makes sense”* (P_S_103) - Very good possibility to increase the level of difficulty (n_I_=2) which is important (n_I_=1) - *“It is nice, that the therapist has influence on my training”* (P_S_103) | - *“Would be nice to have a traffic light to visualize the improvements: red for no improvement, yellow for medium and green for good improvement”* (P_S_115) |
| **Layout/Design/**  **User Interface (UI)** | - Good graphics (n_S_=1) - Logical layout (n_S_=1) |  |
| **Rehabilitation cockpit as a communication tool** | - Liked the idea (n_S_=3) - Good to ask in case of doubts regarding the execution of the games (n_I_=1) - *P501: “I could send a message to ask for clarification (…) could also receive a message as a stimulus and reminder, because sometimes I don´t feel like exercising and the HPs knows the person and works accordingly”* (P_I_501) | - Video communication useful for supervision or explanations (n_I_=3) - Audio call preferable because video might be distracting (n_I_=2) - A chat section would be nice (n_I_=2), especially in combination with timetables telling when the HPs is available (n_I_=1) - Could be useful to create a community/a social platform where the patients not only can talk to the operator but also with each other (n_S_=1) |

Note. n_S_: number of statements of participants from Switzerland, n_I_: from Italy, n_C_: from Cyprus
