# Supplementary material for: A Newly Developed Exergame-Based Telerehabilitation System for Older Adults: Usability and Technology Acceptance Study
Source: JMIR Hum Factors. 2023 Dec 7;10:e48845. doi: 10.2196/48845 (PMC10739244; doi:10.2196/48845)
Supplement: Multimedia Appendix 3 [file humanfactors_v10i1e48845_app3.docx]

## **Multimedia Appendix 3: Performance of older adults in games and assessments**

|  | **Switzerland** | | **Cyprus** | | **Italy** | | **Total** | |
| --- | --- | --- | --- | --- | --- | --- | --- | --- |
|  | Mean | SD | Mean | SD | Mean | SD | Mean | SD |
| Targets points | 53.9 | 26.6 | 27.7 | 12.9 | 30.5 | 15.3 | 37.3 | 22.2 |
| Targets hits | 26.6 | 12.1 | 14.3 | 7.4 | 15.6 | 8.7 | 18.8 | 10.9 |
| Targets misses | 7.5 | 4.4 | 6.9 | 5.1 | 9.7 | 8.7 | 8.0 | 6.3 |
| Tetris points | 199.7 | 151.7 | 93.1 | 71.0 | 95.2 | 78.8 | 129.3 | 115.9 |
| Rockets average speed | 1.2 | 0.5 | 1.7 | 0.9 | 1.0 | 0.7 | 1.3 | 0.7 |
| Rockets steps | 93.6 | 43.2 | 139.7 | 79.1 | 76.6 | 51.6 | 103.3 | 64.5 |
| Rockets maximum speed | 2.5 | 1.4 | 3.1 | 1.5 | 1.8 | 1.0 | 2.5 | 1.4 |
| Evovle catches | 9.7 | 3.9 | 8.1 | 4.7 | 7.3 | 4.0 | 8.4 | 4.2 |
| Evolve collision | 3.8 | 2.0 | 4.0 | 1.9 | 3.8 | 1.8 | 3.9 | 1.8 |
| Evolve precision | 71.5 | 19.2 | 63.1 | 16.5 | 62.9 | 16.9 | 65.9 | 17.6 |
| Evovle points | 6.6 | 3.6 | 4.5 | 4.8 | 3.9 | 3.6 | 5.0 | 4.1 |
| Simon max sequence length | 5.5 | 1.4 | 2.0 | 0.7 | 3.9 | 1.9 | 3.8 | 2.0 |
| Simon points | 29.5 | 9.1 | 10.2 | 5.1 | 18.2 | 11.3 | 19.3 | 11.8 |
| Stroop level 1: average reaction time [ms] | 1116.0 | 108.1 | 1947.0 | 1621.6 | 1929.4 | 1119.5 | 1664.1 | 1180.2 |
| Stroop level 1: errors | 0.1 | 0.3 | 1.1 | 1.7 | 1.1 | 1.8 | 0.8 | 1.5 |
| Stroop level 2: average reaction time [ms] | 1203.3 | 92.7 | 1624.0 | 624.6 | 1594.2 | 487.9 | 1473.8 | 490.1 |
| Stroop level 2: errors | 0.1 | 0.4 | 1.9 | 3.7 | 0.6 | 1.2 | 0.9 | 2.3 |
| Stroop level 3: average reaction time [ms] | 1292.2 | 267.1 | 1301.6 | 738.0 | 2261.5 | 1365.5 | 1618.4 | 1000.4 |
| Stroop level 3: errors | 0.3 | 0.6 | 7.1 | 10.3 | 1.1 | 1.9 | 2.8 | 6.7 |
| Stroop level 4: average reaction time [ms] | 2425.3 | 828.9 | 2897.3 | 1479.9 | 4891.2 | 3665.7 | 3404.6 | 2521.7 |
| Stroop level 4: errors | 2.0 | 2.3 | 8.4 | 5.8 | 5.7 | 3.7 | 5.4 | 4.9 |
| Coordinated Stability completeness of the path [%] | 74.8 | 15.4 | 47.9 | 24.6 | 45.3 | 22.4 | 56.0 | 24.7 |
| Coordinated Stability difference from ideal path [mm] | 1819.3 | 1119.7 | 1558.6 | 1088.8 | 5620.7 | 12864.8 | 2999.5 | 7547.2 |
| Coordinated Stability path-length [mm] | 2412.0 | 1357.8 | 8397.7 | 21355.5 | 932.8 | 678.6 | 3914.1 | 12509.8 |
