# Supplementary material for: A Newly Developed Exergame-Based Telerehabilitation System for Older Adults: Usability and Technology Acceptance Study
Source: JMIR Hum Factors. 2023 Dec 7;10:e48845. doi: 10.2196/48845 (PMC10739244; doi:10.2196/48845)
Supplement: Multimedia Appendix 4 [file humanfactors_v10i1e48845_app4.docx]

## **Multimedia Appendix 4: Usability Protocol: comments, questions, and observations by healthcare professionals and the investigator**

| **Healthcare Professionals** | | | | |
| --- | --- | --- | --- | --- |
| **Category** | **Positive aspects** | | **Negative aspects, requirements, and suggestions for development** | |
|  | **As patient** | **As HPs** | **As patient** | **As HPs** |
| **Senso in general** | | | | |
| **Orientation on the Senso** | - Good orientation on the Senso (n_C_=2) |  |  |  |
| **Size of the central square** |  |  | - Too small (difficult for people with large feet) (n_S_=1) (n_C_=1) |  |
| **Assessment System** | | | | |
| **User-friendliness** | - The warm-up phase clarifies everything better (than the written instructions) (n_I_=1) | - Navigation easy and intuitive (n_S_=2) (n_I_=4) - UI user-friendly and easy to understand (n_S_=3) (n_I_=1) - HPss seemed confident to guide through these assessments by themselves (n_S_=5) - Repetition of the warm-up is a nice feature (n_S_=1) - Parameters provided are ok (n_I_=1) | - Font size of the instructions should be adapted for OAs (n_S_=1) |  |
| **Stroop** | | | | |
| **Content** |  |  | - Not feasible for every patient, should be adapted individually (n_S_=1) (n_I_=1) - Difficult/impossible for older people (n_I_=2) and for analphabets and foreigners (n_I_=1) | - Should be split into single tests to individualize the assessments (some patients unable to perform all 4 parts) (n_S_=1) |
| **Instructions** | - Instructions clear (n=3) (n=7) |  | - Unclear in level 3 and 4 (n_C_=1) (n_I_=3) - Participant got confused (n_C_=2) |  |
| **Performance** |  |  | - Bit of struggle in the warm-up (n_S_=2) |  |
| **Coordinated Stability** | | | | |
| **Content** |  |  | - Requires movements are challenging (n_C_=3) and unnatural (n_I_=1) - extremely difficult for an old person (n_I_=2) |  |
| **Instructions** | - Good (n_S_=2) |  | - Not fully understood (n_I_=1) | - Helpful to add visual instructions (videos or pictures) (n_I_=1) |
| **Safety** |  |  | - Difficult for patients without the handrail (n_C_=1) (n_S_=1) |  |
| **Assessment Report** | | | | |
| **Content** | - Does not require modifications (n_I_=2) | | - Comparison in age group not relevant (n_S_=1) - “It would be useful for HPss to know how these scores (executive functions, balance, strength, endurance and memory) are calculated.” (P_I_602) - If the patient shows it to a doctor, the report should include other indications that are not related only to Senso: e.g. “speak of this with your doctor”, “other clinical exams would be necessary”, “you can also perform other physical activity to improve your condition”.” (P_I_604) | |
| **Usefullness** | - Training recommendations useful for HPs and patients (n_S_=1) (n_I_=3) | |  | |
| **Comprehensibility** | - Clear, well-arranged, well presented, and self-explanatory (n_S_=4) | | - Unclear term “executive function” (even for physiotherapists) (n_S_=2) - Description of “executive functions” and “Percentile” should be explained in simpler language, perhaps with an example or added into a graphic (n_S_=1) (n_I_=4) - Practical examples for executive functions would push the patient (n_I_=1) | |
| **Motivation** | - Nice positive comments [in the report] like “Your strength is top” (n_I_=1) | | - Percentiles might have a demotivating effect (n_S_=2) - Better just to show improvements of the patient (n_S_=1) | |
| **Senso Flex** | | | | |
| **Set-up** | - Easy (n_S_=4) (n=5) (n_I_=2) | | - Alignment of the mat should be specified (e.g. with an arrow) (n_I_=3) - Set-up difficult for people with knee problems (n_S_=1) - internet connection has to be very stable (is often not the case in older adults´ homes or in clinics) (n_S_=5) - “Not everyone will have enough space for the mat. Perhaps the investigators will need to plan to reorganize the space.” (P_I_604) | |
| **Navigation** | - Worked well (n_S_=4) | | - Long step forward for game selection is not intuitive, better instruction needed (n_I_=1) | |
| **Orientation** |  | | - Difficult (especially backwards orientation) (n_S_=2) - No warning sign when center area of the mat is left (n_I_=1) - Center area of the mat should be marked or embossed (n_C_=4) (n_I_=3) - A border with a tactile texture should be added (n_I_=1) | |
| **Balance and Safety** |  | | - Risk of falls (due to balance problems or performance anxiety) (n_I_=1) - Lateral support needed (e.g., sofa, side chairs or walkers) (n_I_=3) | |
| **Sensitivity** | - Good (n=1) | | - Low (n_S_=4) (n_C_=1) (n_I_=2) | |
| **Exergames** | | | | |
| **Content** | - Very interesting for coordination (n_I_=1) - Augmented feedback given by the sound (n_I_=2) - Good challenge for OAs (n_C_=5) - Good exercise for OAs (n_C_=2) - Liked it (n_C_=3) - Beautiful (n_I_=1) - Funny (n_I_=1) | | - Not all games are good for all OAs (n_C_=4) | |
| **Instructions** | - Good (n_S_=2) - Well understood (n_S_=3) - Clear and/or intuitive in case of Targets (n_C_=5) (n_I_=1), and Evolve (n_C_=5) (n_I_=1) | | - Too long (n_I_=1) - Pictures or animations are useful (n_I_=3) - Good familiarization needed (n_C_=2) | |
| **Comprehensibility** |  | | - Simon confusing because the presentation of stimuli happens too fast (n_C_=5) (n_I_=2) | |
| **Performance** | - “The games in the following sessions are certainly facilitated by the fact that the instructions are already known, but in any case, the patients can always amaze you!” (P_I_605) | |  | |
| **Enjoyment and motivation** | - Enjoyed it (n_C_=4) - Will be enjoyed by OAs too despite difficulties (n_C_=2) | | - Unclear aim of some games and no reward (especially Rocket)) (n_I_=2) | |
| **Safety** | - Rocket: Red bar is helpful to prevent patients from running (n_S_=2) | | - Walking on spot (Rocket) is an unnatural type of walking (n_I_=1) and not good for OAs with mobility or heart rate problems (n_C_=1) or for Parkinson patients (n_I_=1) | |
| **Rehabilitation Cockpit** | | | | |
| **Usefulness** | - Useful (n_S_=1) - Training control is immediately transferred to the patient (n_S_=1) - Nice overview (e.g., with the graphs) of the patient’s progress (n_S_=1) which is useful feedback to patients and HPs (n_I_=1) | | - Not every HPs likes to work remotely (n_S_=1) - Monitoring unnecessary or even contra productive (n_S_=1) (most patients don´t like to be monitored all the time (n_S_=1)) | |
| **Layout/Design/**  **UI** | - Very nice (n_I_=1) due to nice colors (n_I_=1), an intuitive UI (n_I_=1), simple and intuitive setting possibilities (n_I_=3) | |  | |
| **Rehabilitation cockpit as a communication tool** | - Nice feature as communication is essential (n_S_=2) (n_I_=1) | | - “It would be useful for the patient to receive a pre-recorded message after each session informing him/her on how the training went. (…) I would add the possibility to arrange a video call.” (P_I_602) - “It could be helpful to implement a channel of communication (…) for example making a videocall (…) during the training session or add a chat for messages, comments, or indications for the patient.” (P_I_603) - “It is advisable to have the possibility of audio-video connection in real time. If this is not possible, it is advisable to allow the HPs to send voice messages to the patient.” (P_I_604, P_I_605) | |

Note. n_S_: number of statements of participants from Switzerland, n_I_: from Italy, n_C_: from Cyprus
